# Intelligent Multimodal Framework for Human Assistive Robotics Based on Computer Vision Algorithms

**DOI:** 10.3390/s18082408

**Published:** 2018-07-24

**Authors:** Eugenio Ivorra, Mario Ortega, José M. Catalán, Santiago Ezquerro, Luis Daniel Lledó, Nicolás Garcia-Aracil, Mariano Alcañiz

**Affiliations:** 1 Institute for Research and Innovation in Bioengineering, Universitat Politècnica de València, 46022 Valencia, Spain; mortega@i3b.upv.es (M.O.); malcaniz@i3b.upv.es (M.A.); 2 Biomedical Neuroengineering Group, Universidad Miguel Hernández de Elche, 03202 Elche, Spain; sezquerro@umh.es (S.E.); llledo@umh.es (L.D.L.); nicolas.garcia@umh.es (N.G.-A.)

**Keywords:** 3D object detection and pose estimation, assistive robotics, eye-tracking, human–computer interface

## Abstract

Assistive technologies help all persons with disabilities to improve their accessibility in all aspects of their life. The AIDE European project contributes to the improvement of current assistive technologies by developing and testing a modular and adaptive multimodal interface customizable to the individual needs of people with disabilities. This paper describes the computer vision algorithms part of the multimodal interface developed inside the AIDE European project. The main contribution of this computer vision part is the integration with the robotic system and with the other sensory systems (electrooculography (EOG) and electroencephalography (EEG)). The technical achievements solved herein are the algorithm for the selection of objects using the gaze, and especially the state-of-the-art algorithm for the efficient detection and pose estimation of textureless objects. These algorithms were tested in real conditions, and were thoroughly evaluated both qualitatively and quantitatively. The experimental results of the object selection algorithm were excellent (object selection over 90%) in less than 12 s. The detection and pose estimation algorithms evaluated using the LINEMOD database were similar to the state-of-the-art method, and were the most computationally efficient.

## 1. Introduction

Approximately 80 million people in the European Union (one-sixth of its population) have a disability. The percentage of people with disabilities is set to rise as the EU population ages [1].

Accessibility is a basic right for all persons with disabilities according to Article 9 of the United Nations Convention on the Rights of Persons with Disabilities. The purpose of accessibility is to enable persons with disabilities to live independently and to participate in all aspects of life. Assistive technologies help all persons with disabilities to improve their accessibility in all aspects of their life. Current trends in assistive technology for supporting activities of daily living (ADL), mobility, communication, and so on are based on the integration of the capabilities of the user and the assistive technologies. The improvement of the interaction and cooperation between the user and the assistive technologies can be split into three main areas: (1) improvements of the assistive devices, such as mechanical parts, electronic parts, etc.; (2) improvements of the user–technology interface; and (3) improved shared control between the user and the assistive technology. The AIDE European project contributes to improving current assistive technologies by developing and testing a modular and adaptive multimodal interface customizable to the individual needs of people with disabilities as well as a totally new shared control paradigm for assistive devices that integrates information from the identification of the residual abilities, behaviours, emotional state, and intentions of the user on one hand and the analysis of the environment through the use of cameras and context factors on the other. This paper describes the computer vision algorithms part of the multimodal interface developed within the AIDE European project.

There are some examples of multi-modal architectures for the interaction and control of assistive robotic devices. Specifically, Meng et al. presented a non-invasive brain–computer interface (BCI) for controlling a robotic arm to complete reach-and-grasp tasks [2]. They used a Microsoft Kinect Motion Sensor to locate and send the position of a target to the robot control system. Another interesting recent paper presented an assisted feeding strategy that uses a Kinect camera and a modular robotic arm to implement a closed-form system that performs assisted feeding [3]. In contrast to these works, our approach uses two cameras (one looking at the environment in front of the user and the other looking at the user) to locate real objects and the user’s mouth position, respectively. In addition, the user wears a pair of eye-tracking glasses to recognise the object at which the user is looking. There are also some works using invasive BCI systems in people with long-standing tetraplegia to control a robotic arm to perform three-dimensional reach and grasp movements [4,5]. Some works can be found in the literature reporting the control of an arm exoskeleton using multimodal interfaces. Specifically, Pedrocchi et al. developed a novel system composed of a passive arm exoskeleton, a robotic hand orthesis, and a neuromuscular electrical stimulation system driven by residual voluntary muscular activation, head/eye motion, and brain signals in the framework of the MUNDUS project [6]. In addition, Frisoli et al. presented a robotic-assisted rehabilitation training with an upper limb robotic exoskeleton for the restoration of motor function in spatial reaching movements [7]. Then, they presented the multimodal control of an arm–hand robotic exoskeleton to perform activities of daily living. The presented system was driven by a shared control architecture using BCI and eye gaze tracking for the control of an arm exoskeleton and a hand exoskeleton for reaching and grasping/releasing cylindrical objects of different size in the framework of the BRAVO project [8]. Most recently, Clemente et al. presented a motion planning system based on learning by demonstration for upper-limb exoskeletons that allow the successful assistance of patients during activities of daily living (ADL) in an unstructured environment using a multimodal interface, while ensuring that anthropomorphic criteria are satisfied in the whole human–robot workspace [9]. In contrast to the previous works, the AIDE multimodal control interface predicts the activity that the user wants to perform and allows the user to trigger the execution of different sub-actions that compose the predicted activity, and to interrupt the task at any time by means of the hybrid control interface based on a system combining gaze tracking, electroencephalography (EEG), and electrooculography (EOG).

Most activities of daily living require complete reach-and-grasp tasks. The grasping task is a common operation for fixed manipulators in a controlled environment, but assistive robotics have the complexity that this environment is not fixed. Moreover, it should be solved in real-time in order to be comfortable for humans and sufficiently precise to perform successful grasps of a variety of objects. To sum up, a grasping task in multimodal assistive robotics requires the processing of a precise location and orientation of common textureless objects in real-time. Some authors have solved it using commercial tracking systems like Optitrack© or ART Track© [10,11,12], but these solutions require the modification of the objects by adding specific marks. Our proposal employs a computer vision approach that does not have that limitation. There are multiple technical approaches to solving this problem, and despite the great advances made recently in the field of computer vision (especially with the new deep learning techniques), it is still a difficult problem to solve effectively—specifically when the 3D object is textureless. For well-textured objects, several methods based on appearance descriptors like SURF or SIFT [13] can be employed to solve this problem. However, most common objects in our context (home) are textureless.

Considering the technical features required, these methods should be efficient, accurate, scalable, and robust to changes in the environment (no controlled light conditions or occlusions).

The main lines of investigation in the field of 3D textureless object pose estimation are methods based on geometric 3D descriptors, template matching, deep learning techniques, and random forests.

Methods based on geometric 3D descriptors employ the information extracted from the geometry of the 3D models of the objects. There are two kinds of 3D descriptors: local descriptors and global descriptors.

On one hand, local descriptors are obtained from characteristic local geometric points from the model. Once descriptors are calculated from the model and from the depth image from the RGB-D camera, a matching correspondence can be obtained. The last stage is usually a refinement of the pose using an iterative closest point algorithm (ICP [14] Among these methods [15] stand out FPFH [16], PPF [17], and SHOT [18]. These methods are very computationally expensive (need several seconds for estimating the object pose), but are robust to occlusions. One of the most employed methods is the point pair features (PPFs). This method was developed by Drost et al. [17] and employs the depth image for estimating local descriptors using normals of the object. Later, several authors have optimised the original implementation, greatly reducing the computational cost of the algorithm (Stefan et al. [19] still requires between 0.1 and 0.8 s for processing an image). However, the algorithm is still too heavy for real-time use.

On the other hand, global descriptors encode the shape of the 3D model in a single vector. The main global descriptor classes are VFH [20], OUR-CVFH [21], and ESF [22]. In contrast to the local descriptors, and as the main disadvantages, it is necessary to first have the 3D reconstruction of the object, as well as to segment the scene before estimating the pose of the object. In addition, these methods are very sensitive to the occlusion of the object. On the other hand and as a main advantage, they are computationally efficient. These types of descriptors are usually used for their efficiency in problems of classification or 3D model retrieval. In addition, note that these geometric methods (both local and global descriptors) can also use the colour information of the object (if it is available), increasing the robustness of the method (e.g., the local descriptor CSHOT [23]).

Methods based on template matching efficiently search through the generated set of templates of a 3D model employing a sliding window approach to find the most similar template within an image, as well as its 2D location using a similarity criterion. Once the most similar template is determined within the image, the initial pose of the 3D object is inferred from the one associated with the template. Within these methods, the algorithm LINEMOD++ [24] stands out. This algorithm is one of the most-employed algorithms by the scientific community for estimating the pose due to its high efficiency and robustness. Specifically, Hinterstoisser et al. [24] were the first to use this LINEMOD detection method to estimate the pose of objects. The LINEMOD method uses the information extracted from the gradients of a colour image and the surface normals. This information is subsequently quantified so that the search for the most similar template is carried out efficiently. Then, Hinterstoisser et al. [24] added a post-process stage in order to strengthen the detection method by eliminating some of the possible false positives. The last stage is a refinement of the pose using an ICP algorithm. This implementation was enhanced by Kehl et al. [25] to increase its scalability.

Another similar template method was proposed by Hodaň et al. [26]. Unlike the original LINEMOD++ method, they initially limit the search to certain areas of the image, by means of a simple filtering technique. The matching between the templates and the remaining possible locations within the image is done with a voting procedure based on hashing. To refine the pose, they use particle swarm optimization (PSO) [26] in place of the ICP algorithm. Following the same line, Zhang et al. [27] proposes that in addition the detection method be invariant to scale, consequently reducing the number of templates on which to perform the search. Despite all these modifications, these methods [25,26,27] are less precise and more computationally expensive than the LINEMOD++ method. The main limitation of methods based on templates is that they are very sensitive to object occlusion. On the contrary, they are usually computationally efficient methods when compared with methods based on 3D descriptors or deep learning techniques.

Recently, multiple methods have appeared that address the problem of estimating the pose of a 3D object through the use of deep learning techniques. Among the most popular methods are the SSD-6D [28], BB8 [29], Pose-CNN [30], and [31,32] methods. The main advantages of these methods are that they allow estimation of the pose using only the RGB sensor information, the scalability, and the robustness against occlusion. However, and as one of the main disadvantages, most of these methods need a large amount of training images to detect and estimate the pose of an object. Moreover, there is the added difficulty of estimating the actual pose associated with the training images. This supposes a great effort and time of work on the part of the user, as much the compilation of images of the 3D model as the estimation of the pose in each of them. To solve this type of limitation, methods such as the SSD-6D [28] method have been used to estimate the pose of objects using deep learning techniques using only synthetic images extracted from the original 3D model. However, these methods can have problems when there are substantial differences between the appearance of the synthetic images of the 3D model and the appearance of the images captured by the camera [28] (e.g., local changes in the illumination due to specular reflections). In turn, simply the change in the specifications of the capture sensor in the test phase can substantially influence the results [33]. To mitigate this problem, it is necessary to obtain 3D models of 3D objects with photorealistic quality. Although these methods present promising results, the 3D models of the objects must have colour information so that the pose can be detected and estimated correctly. This is a problem because it is common to only have access to a CAD model of the object without colour, or models are obtained through the use of a depth camera/RGB-D and KinectFusion technology [34], resulting in non-photorealistic models. It is importand to remark that training these models requires high-end equipment and/or a lot of time once the training information is ready. On the other hand, except for the SSD-6D [28] method that works at 10 fps, these are very computationally expensive, preventing their use in real-time. In addition, all these methods need a high-performance GPU.

Finally, the last types of methods are those based on forest classifiers. Some examples of these methods are those proposed by Brachmann et al. [35,36] in which they predict the 3D coordinates of an object as well as the labels assigned to each class by means of a random forest. Then, they use the RANSAC algorithm to estimate the initial pose. This method is very robust to the occlusion problem. Another outstanding work is the method of Tejani et al. [37]. They use the “latent-class Hough forest” method with the extracted information (features) of the LINEMOD algorithm on RGB-D patches to estimate the pose of the object. This method is invariant to scale and also allows estimation of the pose of multiple instances of the same 3D object.

In summary, this paper presents the computer vision algorithms developed in the AIDE multi-modal architecture for human assistive robotics that is able to give accessibility to persons with disabilities. The main contribution of this computer vision component is the integration with the robotic system and with the other sensory systems (EOG and EEG). The technical achievements solved are the algorithm for the selection of objects using the gaze and especially the state-of-the-art algorithm for the efficient detection and pose estimation of textureless objects. These algorithms were tested in real conditions with patients, and were also thoroughly evaluated both qualitatively and quantitatively. This paper is organised as follows. Section 2 presents the experimental setup with the multi-modal interface composition, the integration with the robotic system, and the developed computer vision algorithms. Section 3 shows quantitative and qualitative experimental results to evaluate the computer vision algorithm, and finally, Section 4 presents the conclusions and the future work planned.

## 2. Materials and Methods

### 2.1. Experimental Section

**Figure 1:**
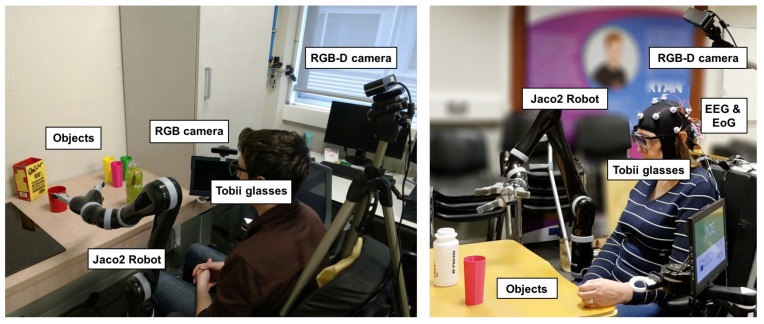
AIDE system integrates three different hardware modules: (i) a full-arm robotic exoskeleton or a Jaco2 robot; (ii) multimodal interfaces, consisting of a pair of gaze-tracking glasses (Tobii glasses) and a hybrid brain–computer interface (BCI) based on electroencephalography (EEG) and electrooculography (EOG); and (iii) context recognition sensors: a RGB-D camera to locate the object’s pose and a camera to compute the user’s head and mouth pose.

All participants were sitting in an electric wheelchair in front of a desk. Moreover, a Jaco2 robot is attached to the wheelchair (see Figure 1). In addition, the multimodal interface is composed of: (1) a pair of gaze-tracking glasses and a hybrid brain–computer interface (BCI) based on electroencephalography (EEG) and electrooculography (EOG); (2) context recognition sensors: two cameras to locate the object’s position and the user’s mouth position; (3) sensors for the monitoring of physiological parameters (breathing rate, heart rate, heart rate variability, galvanic skin response); and (4) a central server (YARP) for the communication. YARP stands for Yet Another Robot Platform. The experiments and results presented in this paper focused on the algorithms used for gaze-tracking and context recognition.

### 2.2. Calibration Methods Robot <-> RGB-D Camera

**Figure 2:**
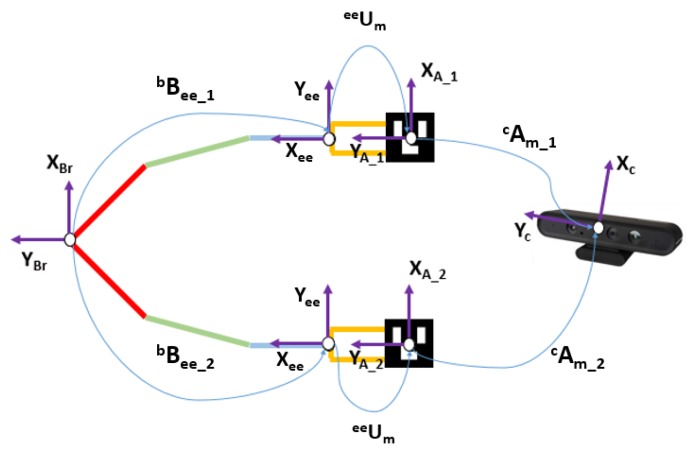
Schematic of the robot—camera problem.

**Figure 3:**
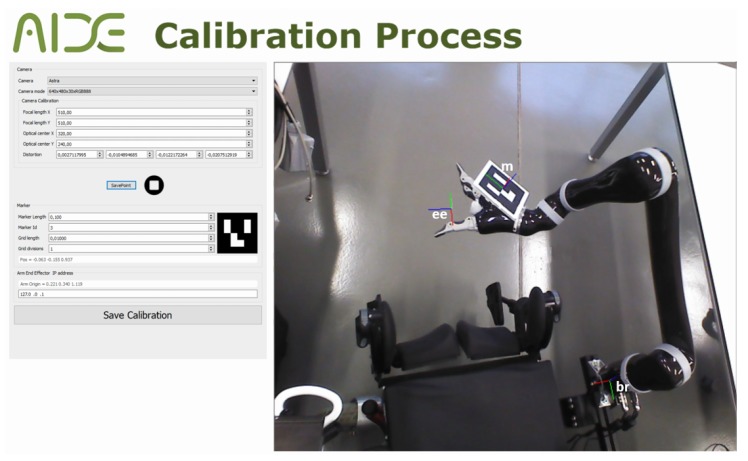
Calibration program.

The objective of the calibration between the RGB-D camera and the robot is to make it possible to transform the coordinates system from the camera to the coordination system of the robot. This problem in robotics is known as *hand–eye calibration*. Specifically, it consists of estimating the homogeneous rigid transformation between the robot hand, or end-effector, to the camera as well as to the world coordinate system (see Figure 2). In the developed platform, the world coordinate system coincides with the robot base and the camera is not in the final effector of the robot but in a fixed position outside the robot. Let the rigid transformation of the robot-base to the end-effector be ${}^{b}B_{ee}$, and ${}^{c}A_{m}$ be the transformation of the camera to an augmented reality mark system. This system is an Aruco [38] mark mounted on a known pose on the robot thanks to a printed piece as can be seen in Figure 3. The transformation ${}^{ee}U_{m}$ between the mark and the robot end-effector is calculated using the CAD schematics of the robot and the printed piece. Thanks to this, the position and orientation of the end-effector can be expressed regarding the robot base and the camera system as shown in Equation (1). From this equation (**Direct Calibration**), the direct transformation ${}^{b}T_{c}$ can be easily extracted.

However, due to inaccuracies in the measurements and transformations obtained from the robot kinematics, Aruco detection, and *U* transformation, the following four optimisation methods were employed to increase the accuracy.

1. **Standard Calibration:** The implementation of the shape registration method in C++ [14].
2. **XS Calibration:** The c1 method of Tabb et al. [39].
3. **XS2 Calibration:** The c2 method of Tabb et al. [39].
4. **Ransac Calibration:** The OPENCV library implementation in C++ of the random sample consensus method (RANSAC optimization).

Methods 1 and 4 employ the strategy of estimating the transformation between a cloud of 3D points expressed in the robot base and a cloud of the same points expressed in the camera system. Methods 2 and 3 were developed by Tabb et al. [39], and are based on the homogeneous matrix equation AX=ZB where *Z* is the transformation from camera to robot base and *X* is the transformation from robot base to world coordinate. The difference between both methods is the cost function employed for the optimisation of transformations, as shown in Equations (2) and (3), respectively.

(1) $${(}^{b}B_{ee}{⟷}^{c}A_{m}{*}^{m}U_{ee})$$

(2) $$c1=\sum_{n-1}^{i=0}{\left|\left|A_{i}X-ZB_{i}\right|\right|}_{F}^{2}$$

(3) $$c2=\sum_{n-1}^{i=0}{\left|\left|A_{i}-ZB_{i}X^{-1}\right|\right|}_{F}^{2}$$

### 2.3. Eye-Tracking Detection

The hardware selected for this task was the Tobii© Pro Glasses 2. This hardware is a mobile lightweight gaze tracker recording both point-of-regard and scene in front of the subject. The gaze point data are sampled at 100 Hz with a theoretical accuracy and Root Mean Square (RMS) precision of 0.5${}^{\circ }$ and 0.3${}^{\circ }$, respectively [40]. This device has two main components: head unit and recording unit. The head unit is a glasses-shaped device with a full-HD RGB camera with a frame rate of 25 fps. The Tobii© Pro Glasses recording unit can record to a Micro-SD (not used in this project), and has battery support and two network interfaces (wireless and Ethernet). A C++ library was developed that receives the video streaming of the glasses and the synchronized gaze point. No Tobii© SDK or proprietary software was employed for this project. The developed software can configure the glasses to work at different image resolutions, set-up frame rates (until 25 fps), and transmits via wireless or Ethernet connection. For this application, only the wireless connection was employed due to some issues detected during the integration phase of the project. Specifically, Tobii© Glasses internally implement a UDP broadcast and an IP6 discovering devices protocol which is incompatible with the YARP system. Gaze information is received in datagram ASCII. code via UDP protocol, and the streaming video is encoded in H264 (also received using a UDP protocol).

The gaze position obtained from the Tobii© Glasses is enhanced using a median filter, obtaining a more stable gaze point. In addition, due the higher acquisition rate of the gaze position than the RGB camera (100 Hz vs. 25 fps), the median filter allows the filtered gaze position to be synchronized with the RGB image.

A deep learning method called YOLOV2 [41] in combination with the gaze point gives us the initial detection of the desired object. There are other deep learning methods to detect objects, such as Faster-RCNN [42] or SDD [43]. However, YOLOV2 was chosen due its great efficiency and robustness in real-time. Specifically, YOLOV2 was trained with the COCO image database [44], which has 91 classes from the YOLOV2 . These classes cover most of the desired objects to manipulate (e.g., glasses, cutlery, microwave, etc.) in this project. Moreover, in the event that a desired object was not in the dataset, it could been trained. Finally, as a result of this stage, the class of the user-selected object is sent to the object detection and pose estimation stage.

### 2.4. Detection and Pose Estimation

The method developed for the detection and pose estimation was derived from the detection method of Stefan et al. [24], known as LINEMOD. The eye-tracking stage gives the ID of the object to track, so the Hinterstoisser et al. algorithm [24] only has to search one class of model. Consequently, it is more efficient, has a lower rate of false positives, and removes the scalability problem of different classes of objects that the Hinterstoisser et al. algorithm experiences.

The LINEMOD method starts with 2D images (colour and depth) synthetically rendered from different points of view and scales of the object 3D model. Viewpoints are uniformly sampled around the object, like going over a virtual sphere with the object in its center. For each of the viewpoints, a set of a RGB-D images and the virtual camera pose $\left\{R,t\right\}$ are saved. Then, a vector of distinctive points, as well as their associated descriptors, are calculated using the RGB-D information, as described in Hinterstoisser et al. [24]. This method defines a template as $V=({\left\{O_{m}\right\}}_{m\in M},\rho )$. *O* is the template feature (surface normal orientation or gradient orientation). *M* is the image information (RGB or depth). ρ is a vector of features locations *r* in the template image. Then, the generated templates are compared in the region of interest (ROI) of the scene image *I* at location *c* based on a similarity measurement over its neighbours ω:

(4) $$\left(I,V,c\right)=\sum_{r\in \rho }\max_{v\in c+\omega }f_{m}\left(O_{m}\left(\omega \right),I_{m}\left(v\right)\right))).$$

This function $f_{m}\left(O_{m}\left(r\right),I_{m}\left(v\right)\right)$ measures the cosine similarity of the features. Then, an empirical threshold is defined based on the score similarity score in order to decide if it is a match. The template matching stage (Equation (4)) was efficiently implemented taking advantage of the SSE instructions of modern CPUs. Furthermore, the detected templates could contain duplicate object instances, so a template clustering algorithm is performed aggregating templates with similar spatial location. However, this detection method can still throw false positives, so as in the original work, the colour information (in the HSV colour space) and the depth information (using an iterative closest point algorithm) were employed to filter these errors. Finally, the pose associated $\left\{R,t\right\}$ with the most similar template was refined with an iterative closest point algorithm—specifically with the *point-to-plane* version.

**Figure 4:**
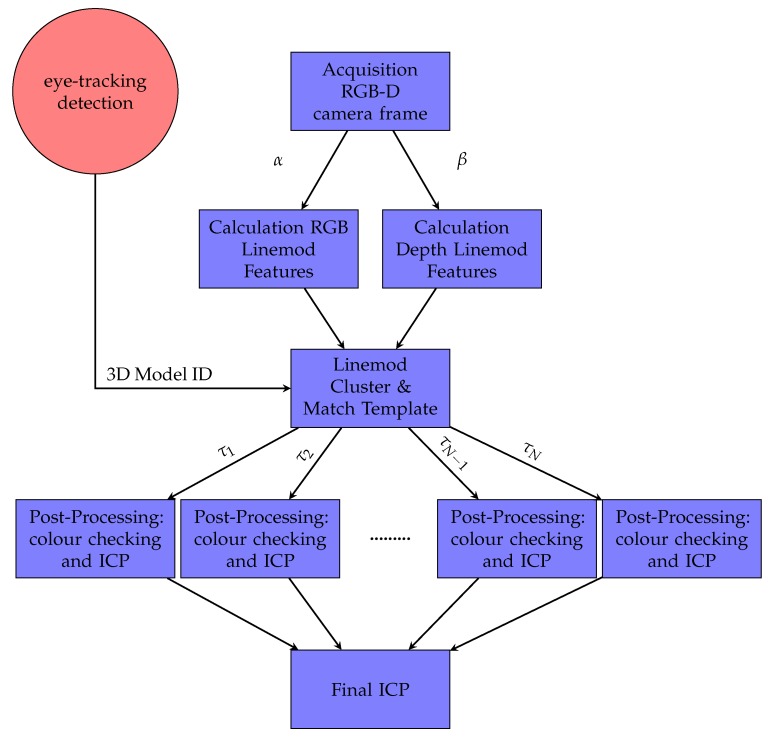
Flow diagram of the LINEMOD++ implemented algorithm. α and β are two independent processes, and τ represents a thread. ICP: iterative closest point.

The main contribution of this part is the optimisation of the LINEMOD detection method [45]. This method was redesigned in order to be multi-processing, so it was split into two independent parts: one process is responsible for extracting the colour information from the RGB image (gradients), while another process is responsible for extracting the depth information (normals from the surface). These processes do not share memory between them so they can be executed in independent physical cores for an optimum performance. Moreover, the post-processing part [24] was also optimised with a multi-threading approach, responsible for eliminating false positives and refining the initial pose obtained. This post-processing part is performed by an ICP algorithm and checking the colour for each of the possible templates in different threads. These threads share memory in order to finish early the execution when one thread finds a valid template. Our method is summarised in Figure 4.

### 2.5. Mouth Pose

The first step in the pose estimation of the mouth is to detect and recognise the user’s face. Face recognition algorithms generally use 2D images for feature extraction and matching [46]. In order to achieve better performance and accuracy, 3D images captured via RGB-D cameras can be employed [47]. Therefore, we decided to employ one for this project. Specifically, we chose the Intel^®^ RealSense™ SR300 RGB-D camera. This camera implements a short-range (maximum 1.20 m) coded light 3D imaging system. The small size of the SR300 provides system flexibility to allow design into a wide range of products.

The mouth pose is obtained using the landmark detection API of the RealSense™ SDK. The algorithm employed returns 78 facial landmark points. For this project, we selected two pupil landmarks and two extreme points of the mouth (left and right). Using these two last 3D points (*L* and *R*) we estimate the mouth pose as follows:

(5) $$Mp=\frac{\vec{LR}}{2}+L.$$

The point Mp is the center point of the mouth and the origin of the mouth pose. We set the axis so that the *x*-axis is in the $\left(\vec{LR}\right)$ direction, *z*-axis is in the direction from Mp to the camera, and the *y*-axis is calculated to be a right-handed coordinate system. In addition, based on the colour information of the detected pupil landmarks, we can estimate if the user is blinking their eyes and know if it is the left or the right eye. For this work, all mouth landmarks were employed to detect when the mouth is open using the area of a convex hull calculated from all of the mouth points provided by the SDK.

## 3. Results

In this section, the results of different experimental sessions to evaluate the methods and/or algorithms reported in this paper are described.

### 3.1. Calibration between Camera and Robot

**Figure 5:**
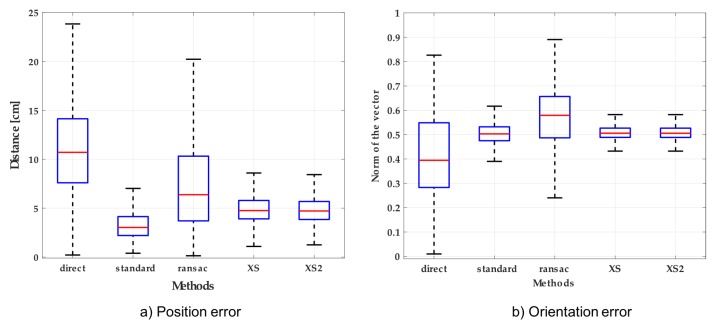
Position and orientation errors using different calibration methods: (**a**) Position error measured as a distance from the correct position; (**b**) Norm of the orientation error vector computed by Rodrigues’ expression.

The position and orientation errors measured using different calibration approaches are shown in Figure 5. The most accurate method regarding position error was the standard method. In the case of orientation error, the most accurate methods were XS and XS2 followed by the standard method. After the evaluation of all the methods, we selected the standard one, which had the best results regarding position error and an admissible accuracy regarding orientation error. Moreover, the comparison of the influence of using different number of calibration points can be found in Appendix A.

### 3.2. Detection and Pose Estimation

In this section, the quantitative results of the detection and pose estimation method are described. These results are compared with the works of other authors. Finally, a detailed analysis of the computational performance of the algorithm was carried out, and as in the quantitative analysis, it is compared with the work of other authors.

#### 3.2.1. Quantitative Validation of the Detection and Pose Estimation of 3D Objects

**Figure 6:**
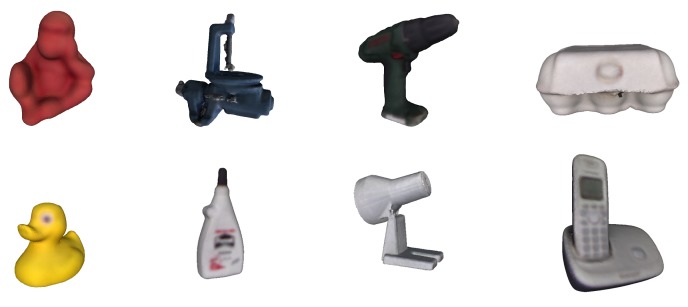
Some 3D models of the LINEMOD dataset.

To quantitatively evaluate the developed method, the LINEMOD dataset [24] was used. Although there are other datasets (e.g., T-LESS [48], Tejani [37], among others), the LINEMOD dataset is undoubtedly the most used by the scientific community to quantitatively evaluate detection and pose estimation methods. The LINEMOD dataset is formed by 15 3D non-textured objects, of which 13 colour 3D models are available (see Figure 6). Each model has a sequence of RGB-D images (around 1200 images in each), in which multiple objects appear from different points of view and distances (in a cluttered environment). Each image has the associated real pose (“ground truth”) of the object and the intrinsic parameters of the RGB-D camera employed for acquiring the image.

The most common metrics employed for comparing the different methods are:

- Average distance (AD): This metric was introduced by Hinterstoisser et al. [24] and is the most employed to quantitatively evaluate the accuracy of pose estimation [19,26,27,28,29,49]. Given a set of vertices of a 3D model, *M*, the actual rotation and translation [R,t] (“ground truth”) and their estimations $\left[\hat{R},\hat{t}\right]$:
  (6) $$m_{AD}=\frac{1}{\left|M\right|}\sum_{x\in M}||\left(Rx+T\right)-\left(\hat{R}x-\hat{T}\right){\left|\right|}_{2}.$$
  when the 3D object is symmetrical, like some of the LINEMOD models (“cup”, “bowl”, “box”, and “glue”):
  (7) $$m_{AD}=\frac{1}{\left|M\right|}\sum_{x_{1}\in M}\min_{x_{2}\in M}\left|\right|\left(Rx_{1}+T\right)-\left(\hat{R}x_{2}-\hat{T}\right){\left|\right|}_{2}.$$
  Traditionally, it is considered that the pose is correct if $m_{AD}\leq k_{m}d,d$ being the diameter of the object, and $k_{m}$ a coefficient ≥ 0. Generally a $k_{m}=0.1$ is used (i.e., 10% of the diameter of the object).
- Shotton criteria (5 cm 5${}^{\circ }$): Using this criteria [24] a pose is considered correct if the rotational error is less than five degrees and the translational error is less than 5 cm. Please note that this metric does not take the size of the object into account.
- 2D Bounding Box: This metric calculates the intersection over union (IoU) [50] between the 2D bounding box obtained by projecting all the vertices of the 3D object with the real pose “ground truth ” in the image and the 2D bounding obtained by projecting all the vertices of the object with the estimated pose. A pose is correct if IoU > 0.5.
- 2D Projections: This metric [36] sets a pose as valid if:
  (8) $$m_{Proj}=\frac{1}{\left|M\right|}\sum_{x\in M}||K\left(Rx+T\right)-K\left(\hat{R}x-\hat{T}\right){\left|\right|}_{2}$$
  is less than 5 pixels. *M* is the set of vertices of the 3D model, *K* is the matrix of intrinsic parameters, $\left[\hat{R},\hat{t}\right]$ is the estimated pose and [R,t] is the true pose. It should be noted that this metric is the most appropriate when you want to estimate the pose of 3D objects in an Augmented Reality system, and so was not used in this work.
- F1-Score. Given PR as true positive, TPR the recall ratio (true positive rate), and PPV the precision ratio (positive predictive value), $F1=2\frac{PR}{PPV+TPR}$ is defined. This metric has been used in References [28,37].

#### 3.2.2. Comparison of the Results with State-Of-The-Art Methods

Firstly, it is worth noting that unlike other authors, quantitative evaluation using the main metrics was carried out in this work. This is of vital importance, since the results obtained often vary substantially depending on the metric employed.

When comparing the results of our method with some of the most popular methods (Table 1) on the LINEMOD dataset and with the AD metric, the results obtained are similar to those of the LINEMOD++ method proposed by Hinterstoisser et al. [24] (95.7% versus 96.6%). This is reasonable since the method developed in this work is based mainly on the LINEMOD++ algorithm. Specifically, the proposed method presents a series of modifications of LINEMOD++ in order to optimise the performance in real scenarios.

Looking in detail at the obtained results (see Table 1 and Table 2) and comparing with the results obtained from other similar works, our method improved upon the results of [17,26,27,35]. It also exceeded by a wide margin the method SSD-6D [28], since it got 76.3% using the RGB information and 90.9% with the RGB-D information compared to the 95.7% obtained in our method. Note also that it improved the accuracy of the method of Brachmann et al. [36] when it only used the colour information (50.2%). In addition, the described method improved the BB8 [29] method with or without refinement of the pose (62.7%).

On the other hand, it matched the results obtained by the method of Zhang et al. [51]. In contrast, the method of Brachmann et al. [36] was more accurate when the depth information was employed in addition to the colour information; specifically, it achieved 99.0% with the AD metric in the LINEMOD dataset.

When comparing with the work of [19], it is worth remarking that they only show the best results of 8 of the 13 3D objects available in the LINEMOD database. Consequently, if we calculate the average obtained using the AD metric of our method for these models, we obtained 96.5% versus 97.8% of the method of Hinterstoisser [19].

Finally, it can be concluded that although more precise methods have appeared in recent years [28] (all of them based on deep learning techniques), especially when the objects are partially visible, our method was not only accurate enough compared to many of the methods in the scientific literature (see Table 1 and Table 2), but it was also (as will be seen in the next section) the fastest of all the methods analysed in this work, allowing pose estimation in real-time with only the requirement of a 3D model (not necessarily with colour) of the 3D object.

**Figure 7:**
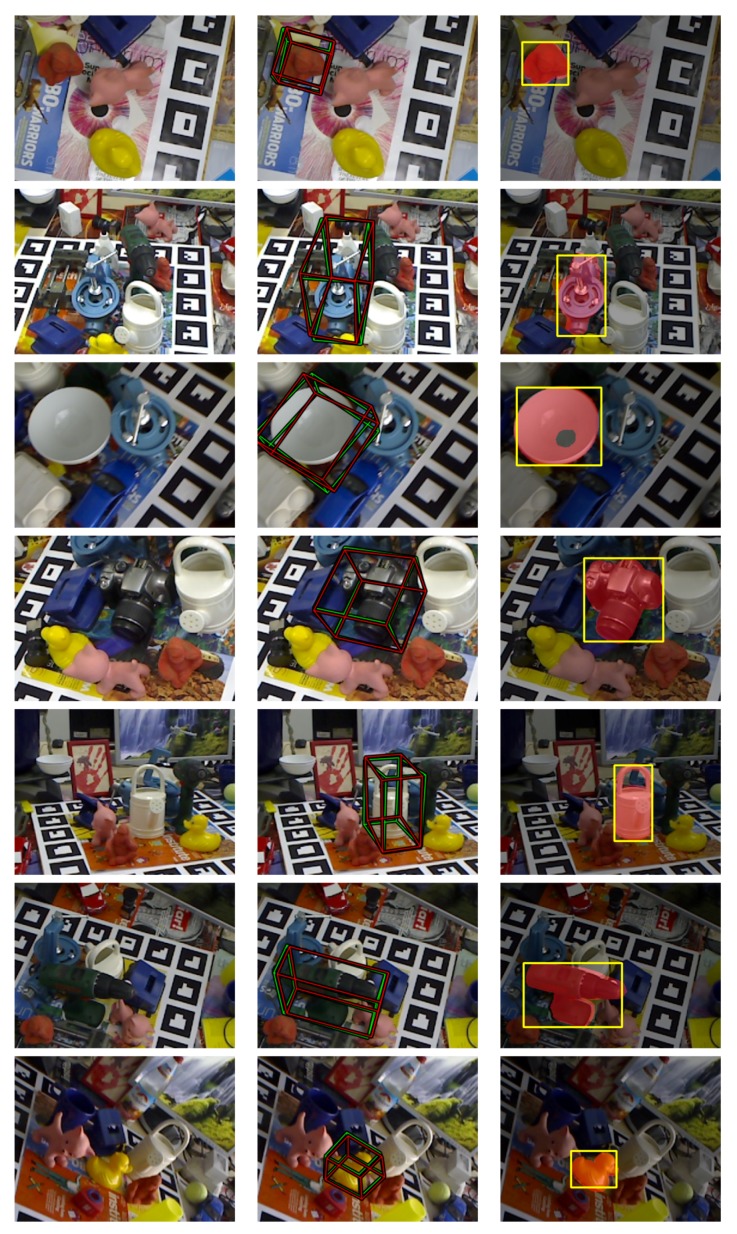
Results visualisation of the estimated pose using the LINEMOD dataset. The bounding box projection of the actual pose is shown in red, and the estimated pose by our method is shown in green (best viewed in colour).

Additionally, Figure 7 shows some qualitative results of the estimated pose using our method in the LINEMOD++ database. Specifically, a projection was done of a bounding box calculated using the estimated pose (in green) and the ground truth pose (in red).

**Figure 8:**
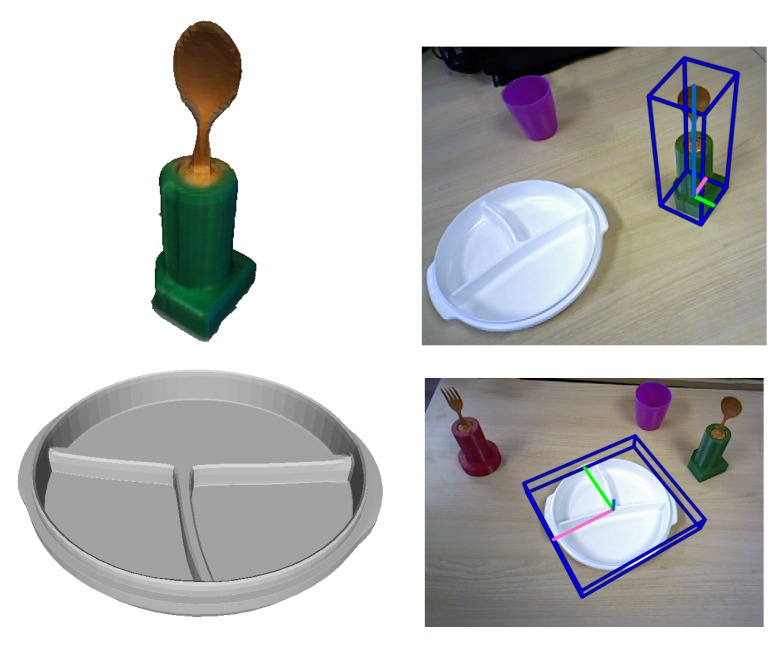
Qualitative results visualisation of the estimated pose of two project objects. The spoon model was scanned and the plate was modelled.

Figure 8 depicts two objects employed in the AIDE project and its pose estimation. Please note that the spoon CAD model was obtained by scanning the actual object using a KinectFusion algorithm [34] and a RGB-D camera, while the plate CAD model was modelled using Autocad 3ds Max. This example sustains the affirmation that our algorithm works with models with poor and high quality.

#### 3.2.3. Computational Cost

One of the objectives of this work was to design a detection and pose estimation system that worked in real-time so that the grasping system could correct the position and orientation of the object. This feature is very interesting in our use case because the user selects the object of interest through an eye-tracking system that works in real time so they can change the objective quickly. In addition, it allows the grasping of moving objects. It is important to note that the performance analysis was done with the limitation that only one object is detected and estimated simultaneously (common in grasping systems).

The developed method had a total computational cost of 0.032 s (31.72 fps) obtained using a battery test of sequences of the LINEMOD dataset, as can be seen in Table 3.

The developed method considerably improved the computational cost in comparison with other similar works. Specifically, it exceeded by a factor of 3× the method on which it is based (LINEMOD++) [24] (see Table 3), obtaining very similar results in precision (see the previous Section 2). This is due to the optimisation performed in the original method. Table 3 shows the times obtained with our parallelized algorithm and without parallelizing. Please note that the tests were performed using the same number of templates as those cited by Hintertoisser et al. [24]. Specifically, 1235 templates were used for each of the 3D models.

In addition, compared with other works (Table 4), our method considerably decreased the computational cost using only the CPU. In more detail, when compared to detection and pose estimation methods based on deep learning techniques [29,31] (with the exception of the SSD-6D method [28] that works at 10 fps), these are very computationally expensive, preventing their use in real-time. Furthermore, all of these methods require a high-performance GPU. In fact, the implemented method exceeded in efficiency the following template matching methods [24,26,27], methods based on geometric descriptors [17,19], and methods based on random forest [36,52,53,54].

Another outstanding aspect is that the implemented method estimates the pose independently in each frame; that is, it does not use tracking techniques such as the one proposed by Tan et al. [55]. This makes it possible in future work to further reduce the computational cost if combined with any of these tracking techniques. It is also important to remark that the obtained time results were calculated after the analysis of the complete image captured by the camera (in this case of size 640×480). However, in our case it was only necessary to analyse the regions of the image obtained from the eye-tracking phase, and consequently the times obtained were further reduced.

The equipment employed for testing our algorithm was a computer with Intel Core i7-7700 (3.60 GHz) with 16 GB of RAM and an Orbbec Astra S RGB-D camera. Our method was implemented in C++ with OpenMP. The optimisation in the detection algorithm was performed in the LINEMOD algorithm implementation of the OPENCV library.

### 3.3. Mouth Pose System

**Figure 9:**
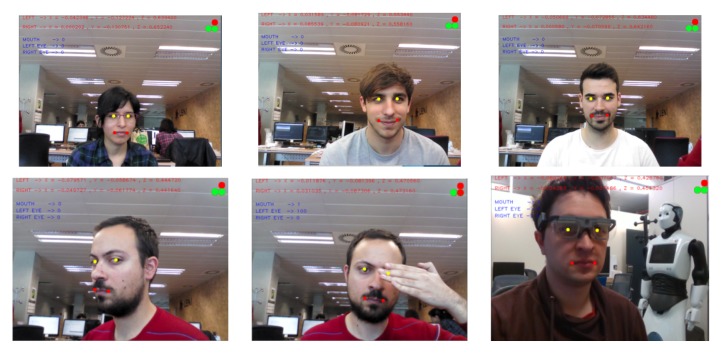
Qualitative results visualisation of the estimated mouth pose with five users. Red points are the mouth landmarks and yellow points the pupil landmarks. The top-right circles indicate if the user has their mouth or the eyes open (green) or closed (red) (best viewed in colour).

The mouth pose algorithm was tested with different users during experimental sessions as can be seen in Figure 9. In this figure, pupil landmarks are coloured yellow while mouth landmarks are red. 3D coordinates are written on the top of the images, and on the top-left corner there are three circle indicators. These indicators change colour to green when the user has their mouth open or if the user is blinking their eyes. These events are also communicated as numerical values and written in blue text on the image. As can be seen, the algorithm worked well with/without facial hair, with glasses, and with different genders.

To assess the stability of the developed method, some extreme positions, partial face occlusion, and wearing an eye-tracking system were tested (shown in the second row of Figure 9).

### 3.4. Eye-Tracking System

To evaluate the performance of object selection using the estimation of gaze point and detection of the type of object already selected, an experiment was conducted with 10 healthy subjects. The experiment consisted of the selection and detection of three kinds of objects (a glass, a bottle, and a fork) wearing the Tobii Glasses. The user had to select the object whose name is shown on a screen in front of the user. The name of the objects appeared randomly, and when the object was selected an audio feedback was provided to the user. The performance of the system was near-excellent since the percentage of average success was 90% and seven out of eleven users only had two or less fails in 20 trials (see Table 5). Regarding the average selection and detection time, the average selection time of all users was around 10 s and the average detection time of all users was around 1 s (see Table 5). Therefore, the users required around 11 s on average to select and detect the object with which they want to interact. Moreover, we measured the angular movements of the neck during the experimental session. The maximum range of motion of each joint was: flexion 15.27${}^{\circ }$, extension 7.5${}^{\circ }$, lateral rotation (right) 68.08${}^{\circ }$, lateral rotation (left) 41.01${}^{\circ }$, lateral flexion (right) 14.54${}^{\circ }$, and lateral flexion (left) 35.86${}^{\circ }$.

### 3.5. Experimental Results

**Figure 10:**
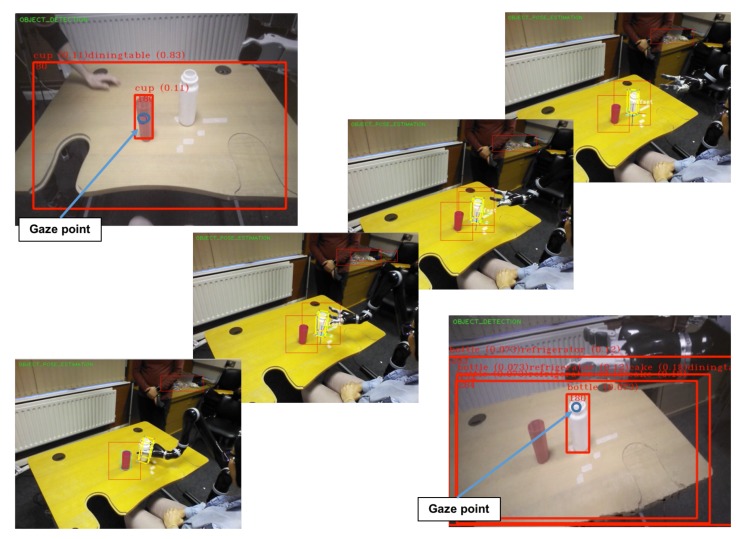
Images of the context recognition and eye-tracking systems in experimental tests. Examples of object detection (cup and bottle) with the estimated gaze position (blue circle). Snapshots of the experiments: grasping a bottle using the Jaco2 robot.

The algorithms and methods presented in this paper were tested in real environments with healthy subjects and subjects with different neurological conditions. The subjects used the hybrid BCI system to trigger the movements of the Jaco2 robot: EEG to control the open/close movement of the gripper and EOG to trigger the movement to grasp the selected object. In Figure 10, some images of the experiments are shown. The performance of the system was very good, and it is out of the scope of this paper to report on the results regarding the use of the hybrid BCI system.

## 4. Conclusions

The AIDE project has developed a complete assistive robotic solution with a novel and revolutionary modular and adaptive multimodal human–machine interface. The computer vision algorithms have an essential role, as has been shown in this manuscript. On one hand, the object selection algorithm is a very useful and natural robot–human interface because the user only needs to stare at the desired object. Furthermore, the complete selection made by the users only costs around 11 s, with an average success of 90% in the test performed. On the other hand, a major contribution presented here is the real-time detection and pose estimation method of textureless objects that allows for precise grasping tasks. As shown in the results, this algorithm outperformed the state-of-the-art in terms of computational cost, with similar precision results to the top methods. A thorough evaluation was made against the popular LINEMOD so that the results can be compared with future methods. Finally, a mouth pose algorithm was employed with the objective of safely operating the robot system. Moreover, the complete assistive robotic system and sensing solution is mounted on a wheelchair, giving a great deal of independence and accessibility to motion-disabled people.

As a future work regarding the detection and pose estimation topic, it is planned to explore a deep learning approach. The main problems of the deep learning methods of pose estimation is that they are not in real-time and it is difficult to obtain the ground-truth data for the training. It is planned to design convolutional neural network that can be computed very quickly, like the YOLO or SSD methods. Furthermore, this model should be able to learn from synthetic generated images. This approach could improve the weaknesses of our method and maintain its strengths.

## Figures and Tables

**Table 1:** Comparison of the results between different detection and pose estimation methods on the LINEMOD dataset [24] using the AD metric and $k_{m}=0.1$, given as the percentage of objects in which the pose was estimated with an error smaller than 10% of the object diameter.

**Sequence**	**Our Method**	**LINEMOD++ [24]**	**Drost [17]**	**Hodaň et al. [26]**	**Brachmann et al. [35]**	**Hinterstoisser et al. [19]**
Ape	97.3%	95.8%	86.5%	93.9%	85.4%	98.5%
Benchwise	95.4%	98.7%	70.7%	99.8%	98.9%	99.8%
Driller	93.0%	93.6%	87.3%	94.1%	99.7%	93.4%
Cam	95.0%	97.5%	78.6%	95.5%	92.1%	99.3%
Can	97.0%	95.9%	80.2%	95.9%	84.4%	98.7%
Iron	98.7%	97.5%	84.9%	97.0%	98.8%	98.3%
Lamp	99.2%	97.7%	93.3%	88.8%	97.6%	96.0%
Phone	97.1%	93.3%	80.7%	89.4%	86.1%	98.6%
Cat	98.8%	99.3%	85.4%	98.2%	90.6%	
Hole punch	92.8%	95.9%	77.4%	88.0%	97.9%	
Duck	99.1%	95.9%	46.0%	94.3%	92.7%	
Cup	97.7%	97.1%	68.4%	99.5%		
Bowl	97.8%	99.9%	95.7%	98.8%		
Box	99.2%	99.8%	97.0%	100.0%	91.1%	
Glue	96.9%	91.8%	57.2%	98.0%	87.9%	
Mean	**95.7%**	**96.6%**	**79.3%**	**95.4%**	**92.5%**	**97.8%**
**Sequence**	**Zhang et al. [27]**	**Kehl et al. [32]**	**Zhang et al. [51]**	**BB8 [29]**	**SSD-6D with RGB-D [28]**	
Ape	96.3%	96.9%	93.9%			
Benchwise	90.4%	94.1%	99.8%			
Driller	95.2%	96.2%	94.1%			
Cam	91.3%	97.7%	95.5%			
Can	98.2%	95.2%	95.9%			
Iron	98.8%	98.7%	97.0%			
Lamp	91.4%	96.2%	88.8%			
Phone		92.7%	92.8%			
Cat	91.8%	97.4%	98.2%			
Hole punch	97.8%	96.8%	88.0%			
Duck	91.8%	97.3%	94.3%			
Cup		99.6%	99.6%			
Bowl		99.9%	99.9%			
Box	99.8%	99.9%	100.0%			
Glue	94.6%	78.6%	98.0%			
Mean	**94.7%**	**95.8%**	**95.7%**	**62.7%**	**90.9%**	

**Table 2:** Results of our detection and pose estimation system on the LINEMOD dataset [24] using different metrics. The percentage is calculated as the number of times that the pose was estimated correctly with respect to the total number of images for each of the sequences. AD: average distance; IoU: intersection over union.

Model	6D Pose (5 cm 5${}^{\circ }$)	6D Pose (AD)	2D Bounding Box (IoU)	F1-Score (AD)
Ape (1235)	98.94%	97.33%	98.86%	0.9864
Bench Vise (1214)	95.46%	95.46%	95.46%	0.9768
Driller (1187)	93.09%	91.24%	93.85%	0.9542
Cam (1200)	95.08%	94.50%	95.17%	0.9717
Can (1195)	97.07%	91.88%	97.07%	0.9577
Iron (1151)	98.70%	98.00%	98.87%	0.9899
Lamp (1226)	99.26%	98.04%	99.26%	0.9901
Phone (1224)	97.11%	97.11%	97.11%	0.9853
Cat (1178)	98.89%	98.89%	98.89%	0.9944
Hole punch (1236)	92.80%	91.35%	92.72%	0.9547
Duck (1253)	99.12%	96.96%	99.12%	0.9846
Cup (1239)	97.74%	97.74%	97.66%	0.9881
Bowl (1232)	97.81%	97.81%	97.81%	0.9889
Box (1252)	99.28%	99.28%	99.28%	0.9963
Glue (1219)	96.97%	90.26%	96.97%	0.9495
Mean	**97.15%**	**95.72%**	**97.20%**	**0.9779**

**Table 3:** Breakdowns of time in seconds of the detection and pose estimation algorithm on each of the sequences of the LINEMOD dataset. The algorithm implemented in the project (multi-core version) improved upon the performance of the LINEMOD++ algorithm by a factor of three [24].

Sequence	Total Time (One-Core)	Total Time (Multi-Core)
Ape (1235)	0.1070	0.0401
Bench Vise (1214)	0.0581	0.0289
Bowl (1231)	0.0748	0.0316
Cam (1200)	0.0646	0.0319
Can (1195)	0.0597	0.0288
Cat (1178)	0.0698	0.0308
Cup (1239)	0.0896	0.0367
Driller (1187)	0.0582	0.0291
Duck (1253)	0.0836	0.0333
Box (1252)	0.0830	0.0344
Glue (1219)	0.0837	0.0335
Hole punch (1236)	0.0831	0.0343
Iron (1151)	0.0621	0.0300
Lamp (1226)	0.0577	0.0287
Phone (1224)	0.0624	0.0288
Mean	0.0731	**0.0320**

**Table 4:** Time comparison (seconds) of different methods for detection and pose estimation.

Method	Time (seconds)	Use GPU
LINEMOD++ [24]	0.12 s	x
Hodaň et al. [26]	0.75 to 2.08 s	√
Brachmann et al. [36]	0.45 s	x
Drost et al. [17]	6.30 s	x
Hinterstoisser et al. [19]	0.1 to 0.8 s	x
Doumanaglou et al. [53]	4 to 7 s	x
Tejani et al. [52]	0.67 s	x
BB8 [29]	0.30 s	√
Zhang et al. [51]	0.80 s	–
Zhang et al. [27]	0.70 s	x
Michel et al. [54]	1 to 3 s	x
Do et al. [31]	0.10 s	√
SSD-6D [28]	0.10 s	√
Ours	**0.03** s	x

**Table 5:** Object selection using the estimation of gaze point and detection of the type of object.

Users	Average Selection	Standard	Average Detection	Standard	Number	Successes	Failures
	Time (s)	Deviation	Time (s)	Deviation	of Trials		
user 1	10.00	13.68	1.02	0.05	20	20	0
user 2	6.38	5.64	1.00	0.02	20	20	0
user 3	18.81	32.52	0.98	0.04	20	20	0
user 4	4.97	2.15	0.96	0.05	20	16	4
user 5	24.63	46.31	0.96	0.05	20	15	5
user 6	6.39	6.98	1.08	0.69	20	18	2
user 7	4.04	1.02	0.96	0.04	20	19	1
user 8	6.05	5.30	1.03	0.03	20	15	5
user 9	14.75	17.32	0.97	0.02	20	18	2
user 10	5.151	1.90	1.06	0.05	20	19	1

**Figure A1:**
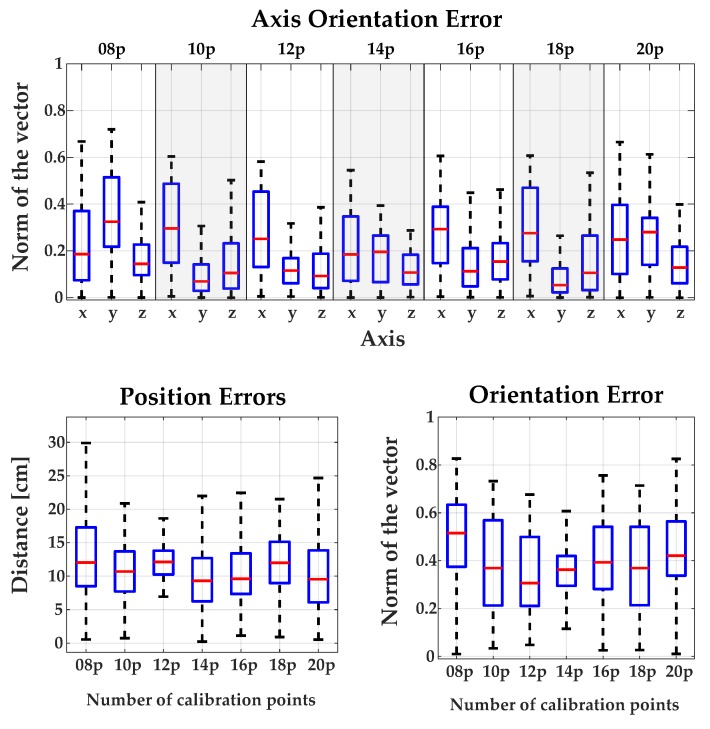
Results obtained by the direct calibration method using different numbers of calibration points.

**Figure A2:**
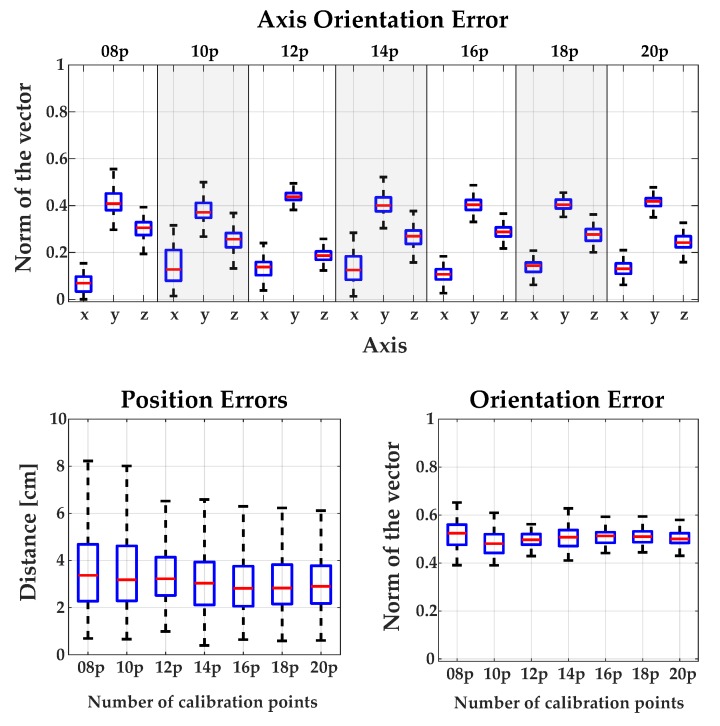
Results obtained by the standard calibration method using different numbers of calibration points.

**Figure A3:**
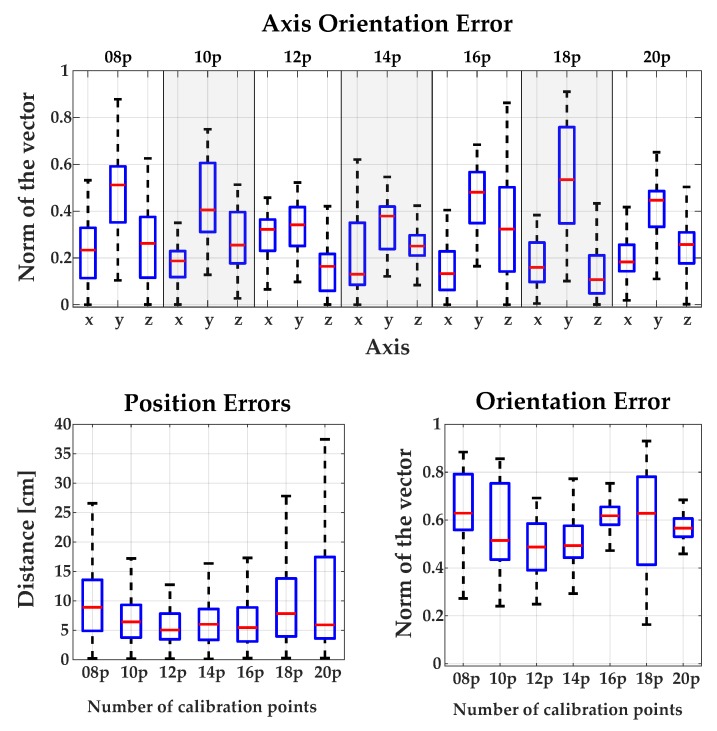
Results obtained by the RANSAC calibration method using different numbers of calibration points.

**Figure A4:**
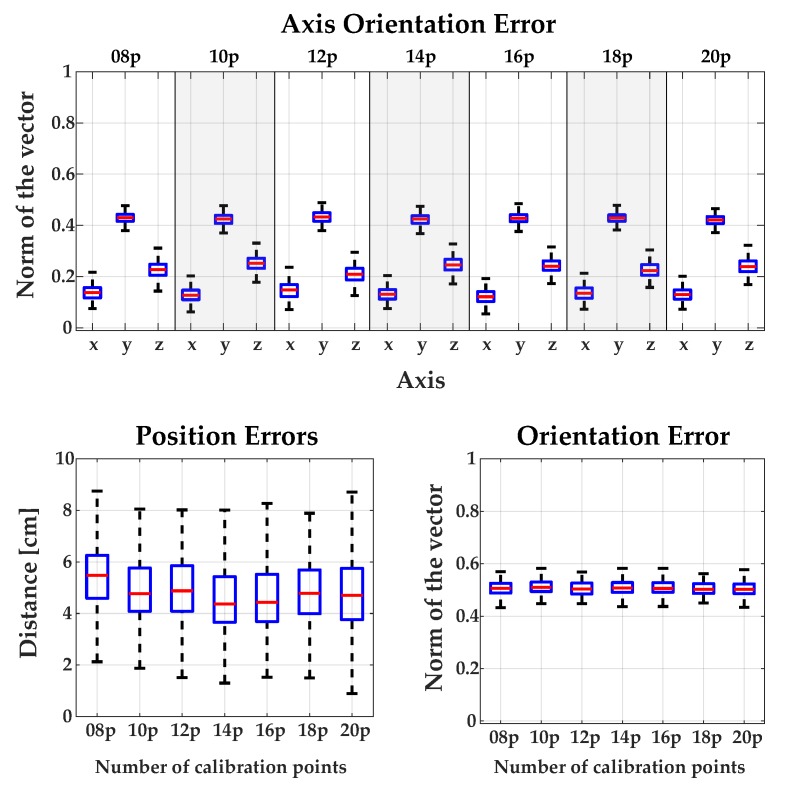
Results obtained by the XS calibration method using different numbers of calibration points.

**Figure A5:**
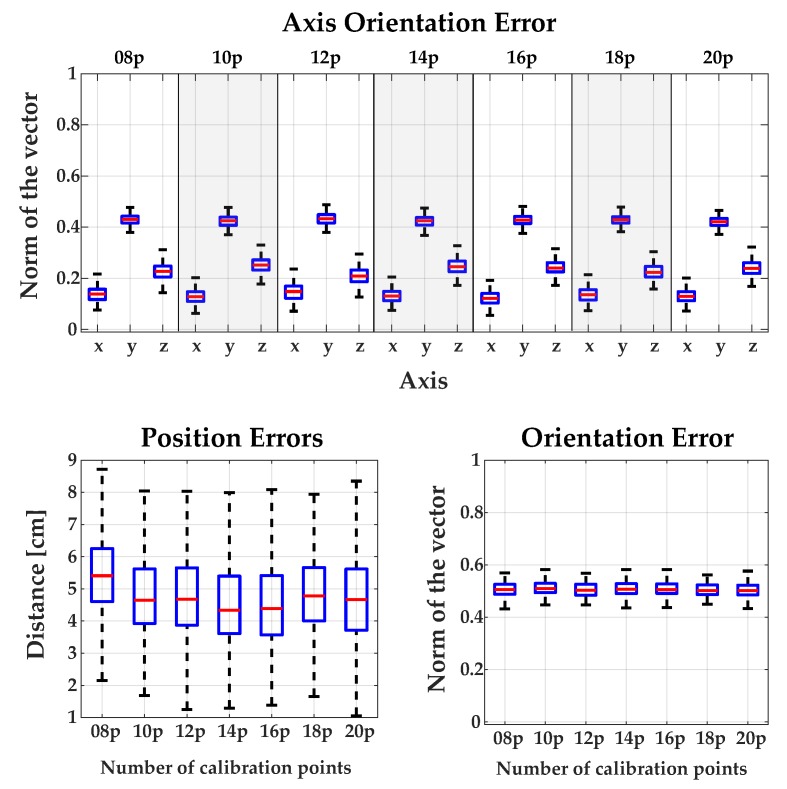
Results obtained by the XS2 calibration method using different numbers of calibration points.
